# Effect of two different preparations of platelet-rich plasma on synoviocytes

**DOI:** 10.1007/s00167-014-3113-3

**Published:** 2014-06-19

**Authors:** Elisa Assirelli, Giuseppe Filardo, Erminia Mariani, Elizaveta Kon, Alice Roffi, Franca Vaccaro, Maurilio Marcacci, Andrea Facchini, Lia Pulsatelli

**Affiliations:** 1 Laboratory of Immunorheumatology and Tissue Regeneration/RAMSES, Rizzoli Orthopaedic Institute, Via di Barbiano 1/10, 40136 Bologna, Italy; 2 Laboratory of Biomechanics and Technology Innovation/NABI, 2nd Orthopaedic and Traumatologic Clinic, Rizzoli Orthopaedic Institute, Via di Barbiano 1/10, Bologna, Italy; 3 Immunohematology and Transfusion Medicine Service, San Pietro Hospital, Via Cassia 600, Rome, Italy; 4 Department of Medical and Surgical Sciences, University of Bologna, Bologna, Italy

**Keywords:** Osteoarthritis, Synoviocytes, Platelet-rich plasma, Soluble factors

## Abstract

**Purpose:**

To analyse the modifications induced by two different platelet-rich plasma (PRP) preparations on osteoarthritis (OA) synoviocytes, by documenting changes in gene expression of factors involved in joint physiopathology.

**Methods:**

OA synoviocytes were cultured for 7 days in medium with different concentrations of either P-PRP (a pure platelet concentrate without leucocytes but with a limited number of platelets), L-PRP (a higher platelet concentrate containing leucocytes) or platelet-poor plasma (PPP). Gene expression of interleukin (IL)-1beta, IL-6, IL-8/CXCL8, tumour necrosis factor alpha, IL-10, IL-4, IL-13, metalloproteinase-13, tissue inhibitor of metalloproteinase (TIMP)-1, (TIMP)-3, (TIMP)-4, vascular endothelial growth factor, transforming growth factor beta1, fibroblast growth factor (FGF)-2, hepatocyte growth factor (HGF), hyaluronic acid (HA) synthases (HAS)-1, (HAS)-2, and (HAS)-3 was analysed by RT-PCR. HA production was determined in culture supernatants by ELISA.

**Results:**

IL-1β, IL-8 and FGF-2 were significantly induced by L-PRP compared to both P-PRP and PPP; HGF was down-modulated by L-PRP versus both P-PRP and PPP, and an inverse dose–response influence was shown for all preparations. Expression level of TIMP-4 was lower in the presence of L-PRP compared with P-PRP. HA production and HAS gene expression did not seem to be modulated by PRP.

**Conclusions:**

L-PRP is able to sustain the up-regulation of proinflammatory factors, (IL-1beta, IL-8 and FGF-2), together with a down-modulation of HGF and TIMP-4 expression, two factors that have been recognized as anti-catabolic mediators in cartilage, thus supporting the need to further optimize the PRP preparations to be applied in clinical practice.

## Introduction

Osteoarthritis (OA) is a common disease that will affect almost half the population at some point in their life through pain and decreased functional capacity [44]. Numerous approaches have been proposed as non-invasive treatments with variable success rates, but none has clearly shown to modify the natural history of the disease and can be considered an ideal procedure for the treatment of joint degenerative processes [29, 33].

Among the new emerging treatment options, injective approaches based on the use of autologous blood derivatives have been introduced into clinical use. In particular, despite the lack of robust literature, there is increasing interest in the local administration of platelet-rich plasma (PRP) to provide a biological stimulus to regeneration [20]. Evidence to support its use has been gathered from the role played in tissue healing, cellular recruitment, growth, maturation and in modulating inflammation by several of the platelet-derived bioactive molecules [13, 46, 57, 58].

Based on this biological rationale, platelet concentrates have been used as a minimally invasive injective treatment for cartilage degeneration and OA for almost a decade [18, 25, 55, 60]. Recently some high-level trials showed overall positive results, thus confirming the potential of this biological treatment approach for knee cartilage degeneration and OA [19, 47, 51]. However, a deeper analysis showed that the published findings were not consistent and sometimes contradictory.

Among the possible explanations for these contradictory findings, we can hypothesize differences in patient selection, treatment application modality and methodological mistakes. However, what surely emerges from the literature analysis is that the main limit is currently the heterogeneity of products used and the lack of knowledge about their mechanism of action and therefore their appropriate indications, and which patients or pathology phase might benefit most from this biological approach. In particular, PRP is defined as a blood derivative with a higher platelet number compared with whole blood before centrifugation and the numerous preparation methods differ not only in platelet concentration, but also in the presence of others cells, such as white blood cells, whose role in the PRP mechanism of action is still controversial [1, 15, 17, 24]. In fact, some authors attribute deleterious effects to the presence of leucocytes in PRP preparation, due to the release of inflammatory mediators, proteases and reactive oxygen by these cells [9, 27]. On the other hand, leucocytes might be considered as a source of cytokines and enzymes that appear to be involved in the infection prevention [43].

The majority of the studies concerning clinical response and in vitro PRP effects on joint cells are concentrated on cartilage tissues [34, 54], while there are currently few studies concerning the effect on synovial tissue (Reviewed in [22]). In the last few years, together with cartilage and bone, a growing body of evidence has highlighted the relevance of synovial tissue as an active player in inducing the progressive OA joint damage, via the release of soluble inflammatory factors that contribute to increasing and perpetuating cartilage damage [26, 37, 52], Therefore, considerable part of the symptomatic improvement obtained with PRP injections might be due to an interaction between the released molecules and the synovial tissue.

Furthermore, majority of the previously reported studies have evaluated the biological effect of PRP up to a maximum of 96 h, and then, long-term investigation on biological effects induced by PRP is needed, in order to address another debated clinical issue relating to the timing of PRP administration.

Bearing in mind these issues, the aim of this study was to analyse the modifications induced by PRP on OA synoviocytes in vitro and document changes in gene expression of an extended panel of molecules implicated in the physiopathology of the joint environment, including inflammatory and anti-inflammatory cytokines, growth factors, extracellular matrix-degrading enzyme and their inhibitors.

Moreover, since the abbreviation PRP includes several heterogeneous products, a secondary aim was to compare the effects of two of the main procedures on synoviocytes, which are already used in clinical practice, based on two PRP preparation approaches that differ both in amount and type of concentrated cells.

Two experimental key points were considered: first, an incubation time point of 7 days was chosen to reproduce the scheduled timing of PRP administration in OA treatment, usually performed according to a series of repeated injections on a weekly basis [19]. Second, to mimic the therapeutic condition in the joint environment, the dilutions of the PRP entire preparations (not only the released supernatant) were allowed to clot directly in the culture plates, by taking advantage from the Transwell™ device to avoid cell–cell contact.

The research hypothesis was that PRP biological effects may be sustained up to 7 days and that the difference in platelet and leucocytes concentration in PRP preparations as well as the use of different PRP amount may lead to different response.

## Materials and methods

Seven healthy men (age range 27–38 years) were enrolled on a voluntary basis to undergo a blood sample collection (200 ml per subject). Exclusion criteria were systemic disorders, infections, smoking, non-steroidal anti-inflammatory drug use 5 days before blood donation, haemoglobin values lower than 11 g/dl and platelet values lower than 150 × 10^3^/μl. Subject anonymity was assured by assigning a code to each sample.

### Preparation of platelet concentrates

PRP was prepared according to two different strategies: a one-spinning procedure, aimed at obtaining a pure platelet concentrate without leucocytes but with a limited number of platelets through one centrifugation, and a two-spinning procedure, aimed at obtaining a higher platelet concentration but with the presence of leucocytes through two centrifugations.

In more detail, for the one-spinning pure PRP (P-PRP) procedure, a 45-ml venous blood sample was divided into five tubes containing 1 ml of trisodium citrate solution (3.8 %) and centrifuged (460*g* for 8 min). Then, 1 ml/tube of the platelet-rich supernatant on the red blood cell pellet was collected, while carefully avoiding leucocyte harvesting [3, 48]. For the two-spinning leucocyte PRP (L-PRP) procedure, a 150-ml venous blood sample was collected in a bag containing 21 ml of sodium citrate and centrifuged at 730*g* for 15 min. Most of the red blood cells were eliminated, and the resulting plasma and buffy-coat were transferred to a separate bag through a closed circuit. After a second centrifugation at 3,800*g* for 10 min, the supernatant was collected to produce PRP. During the second centrifugation platelet, poor plasma (PPP) was also collected and used as the control [48]. The platelet and the white blood cell concentrations were determined by a haematology analyser (COULTER LH 750): linearity was 5–1,000 × 10^3^/μl for platelet count and 0.1–100 × 10^3^/μl for white blood cell count.

Both PRP preparations and PPP were divided into two aliquots, one used for cell culture supplementation and the other one for released factor evaluations.

### Evaluation of factors released from platelet gel

Each sample of PRP and PPP preparations was activated with 10 % CaCl_2_ (22.8 mM final concentration) and incubated for 7 days at 37 °C in 5 % CO_2_, in agreement with cell culture scheduled time point and PRP therapeutic administration in OA [19].

After centrifugation (for 15 min at 2,800*g* at 20 °C), the released supernatant was collected and frozen at −30 °C until used for evaluating interleukin (IL)-1β, fibroblasts growth factor (GF) 2 (FGF-2), hepatocyte GF (HGF), platelet-derived GF AB/BB (PDGF AB/BB), transforming GF (TGF-β1), and vascular endothelial GF (VEGF) concentrations (Standard range IL-1β 28,830.00–1.76 pg/ml; FGF-2 18,336.00–1.12 pg/ml; HGF 37,910.00–2.31 pg/ml; PDGF AB/BB 10,000.00–0.64 pg/ml; TGF-β1 30,019.00–1.83 pg/ml; VEGF 28,440.00–1.79 pg/ml). Samples were assayed in duplicate and factors simultaneously evaluated using commercially available bead-based sandwich immunoassay kits (Bio-Rad Laboratories, Hercules, CA, USA and Millipore Corporation, Billerica, MA, USA) [39]. Intra-assay and inter-assay coefficients of variation were, respectively, estimated between 2–9 % and 5–12 %.

The immunocomplexes formed on distinct beads were quantified by using the Bio-Plex Protein Array System (Bio-Rad Laboratories). Data were analysed by using the Bio-Plex Manager software version 6.0 (Bio-Rad Laboratories). Standard levels between 70 and 130 % of the expected values were considered accurate and were used.

### Cell isolation and culture

Synovial fibroblasts were isolated from patients with OA (*n* = 3 Kellgren–Lawrence grade II–III [32]) undergoing joint surgery. The cells were isolated by enzymatic digestion. Briefly, the synovial tissue was washed twice in phosphate-buffered saline (PBS) and minced into small pieces. Subsequently, two digestions were performed on synovial tissue: the first one with 0.1 % trypsin (Sigma-Aldrich) in PBS at 37 °C, 5 % CO_2_ for 30 min, the second one with 0.1 % collagenase P (Roche) at 37 °C for 1 h under constant rotation. The resulting cell suspension was filtered and centrifuged at 500*g* for 10 min. The cells were seeded into culture flasks and maintained with OPTIMEM (Gibco-BRL, Life Technologies Grand Island, NY, USA) culture medium supplemented with 100 U/ml penicillin, 100 µg/ml streptomycin (Invitrogen, Carlsbad, CA, USA) in a humidified atmosphere at 37 °C with 5 % CO_2_. All experiments were performed on cells obtained between the third and fifth passage.

Subconfluent cultures of synoviocytes were trypsinized, and cell viability was assessed by eosin dye exclusion; the cells were plated at a density of 20,000–25,000 cells/cm^2^ in 12-well tissue-culture plates and maintained with serum-free culture medium (prepared as previously described) for 24 h. Then, culture medium was supplemented with either P-PRP, L-PRP or PPP obtained from each subject (*n* = 7) at 5, 10 or 20 % (vol/vol) previously activated with 10 % calcium chloride (CaCl_2_ 22.8 mM final concentration) to produce a platelet gel and release the granule content. The incubation period was 7 days, during which time the culture medium was not changed. To maintain PRP-activated platelets in contact with synoviocyte monolayers avoiding direct mixing, a Transwell device was used (pore 0.4 μm; Corning, Costar). All experiments were run in parallel. Cell viability was evaluated using the Alamar blue colorimetric assay (AbD Serotec, UK) on day 0, 1, 3 and 7. Briefly, cells were incubated with 10 % Alamar Blue, and after 3 h, the fluorescence was read at 540-nm excitation–590-nm emission wavelength, using a microplate-reader (CytoFluor™ 2350, Millipore, Bedford, MA, USA). Absorbance was directly proportional to the number of living cells in the culture, as indicated by the manufacturer’s data sheet. All cultures utilized for the subsequent experiments showed a number of living cells ≥90 %.

At the end of the incubation time (7 days) culture, supernatants were collected and maintained at −80 °C until their use in ELISA test and synovial fibroblasts were lysed for RNA extraction.

### Synovial fibroblasts gene expression analysis

Samples were assayed with real-time quantitative reverse transcriptase polymerase chain reaction (RT-PCR) for IL-1β, IL-4, IL-6, IL-8/CXCL8, IL-10, IL-13, tumour necrosis factor (TNF)α, VEGF, TGF-β, FGF-2, HGF, metalloproteinase (MMP)-13, tissue inhibitor of metalloproteinase (TIMP)-1, TIMP-3, TIMP-4, hyaluronic acid synthases (HAS)-1, HAS-2, and HAS-3 gene expression.

Total RNA was isolated using TRIZOL reagent (Invitrogen) following the manufacturer’s recommended protocol. RNA was reverse-transcribed using superscript first-strand kit (Invitrogen).

RNA-specific primers for PCR amplification (Table 1) were generated from GeneBank sequences using Primer 3 Software. Real-time PCR was run on the LightCycler Instrument (Roche) using the SYBR Premix Ex Taq (TaKaRa biotechnology), and the increase in PCR product was monitored for each amplification cycle by measuring the increase in florescence due to the binding of SYBR Green I Dye to dsDNA. Technical specifications of light cycler instrument used to perform PCR allow to have a uniform temperature for all samples during each run of PCR, increasing the reproducibility of the data. The crossing point values were determined for each sample, and specificity of the amplicons was confirmed by melting curve analysis. Amplification efficiency of each amplicon was evaluated using tenfold serial dilutions of positive control cDNAs and calculated from the slopes of log input amounts plotted versus crossing point values. They all were confirmed to be high (>92 %) and comparable; mRNA levels for each target gene were calculated normalized to glyceraldehyde-3 phosphate dehydrogenase (GAPDH, reference gene), and according to the ∆∆Ct method, the data were calculated as the ratio of each gene to GAPDH and expressed as “Number of molecules per 100,000 GAPDH”.

**Table 1:** List of primers used in Real-Time PCR

RNA template	Primer sequences (5′–3′)	Annealing temperature (°C)	References
GAPDH	5′-CCTGGCCAAGGTCATCCATG	60	Primer 3
	3′-CGGCCATCACGCCACAGTT		
IL-1β	5′-GTGGCAATGAGGATGACTTGTT	60	Primer 3
	3′-TGGTGGTCGGAGATTCGTAG		
IL-6	5′-TAGTGAGGAACAAGCCAGAG	60	Primer 3
	3′-GCGCAGAATGAGATGAGTTG		
IL-8	5′-CCAAACCTTTCCACCC	60	Primer 3
	3′-ACTTCTCCACAACCCT		
TNF-α	5′-AGCCCATGTTGTAGCAAACC	60	Primer 3
	3′-ACCTGGGAGTAGATGAGGTA		
VEGF	5′-TGATGATTCTGCCCTCCTC	60	Primer 3
	3′-GCCTTGCCTTGCTGCTC		
FGF-2	5′-CGGCTGTACTGCAAAAACGG	60	Primer 3
	3′-TTGTAGCTTGATGTGAGGGTCG		
HGF	5′-ATACTCTTGACCCTCACACC	60	Primer 3
	3′-TGTAGCCTTCTCCTTGACCT		
TGF-β	5′-CAACAATTCCTGGCGATACCT	60	Primer 3
	3′-TAGTGAACCCGTTGATGTCC		
HAS-1	5′-TGGTGCTTCTCTCGCTCTACG	60	[14]
	3′-GAACTTGGCAGGCAGGAGG		
HAS-2	5′-AAATGGGATGAATTCTTTGTTTATG	60	[14]
	3′-GGCGGATGCACAGTAAGGAA		
HAS-3	5′-CAGCTGATCCAGGCAATCGT	60	[14]
	3′-TGGCTGACCGGATTTCCTC		
TIMP-1	5′-CCGACCTCGTCATCAG	60	Primer 3
	3′-GTTGTGGGACCTGTGGAA		
TIMP-3	5′-CCTTGGCTCGGGCTCATC	60	Primer 3
	3′-GGATCACGATGTCGGAGTTG		
IL-4	5′-CAGTTCCACAGGCACAAG	60	Primer 3
	3′-CTGGTTGGCTTCCTTCACA		
IL-13	5′-GCACACTTCTTCTTGGTC	60	Primer 3
	3′-TGAGTCTCTGAACCCTTG		
IL-10	5′-CTTTAAGGGTTACCTGGGTTG	60	Primer 3
	3′-CTTGATGTCTGGGTCTTGG		
MMP-13	5′-TCACGATGGCATTGCT	60	Primer 3
	3′-GCCGGTGTAGGTGTAGA		

TIMP-4 was purchased from Qiagen (Hilden, Germany) (Cat no. PPH00889E)

### Measurement of HA levels

HA in cell culture supernatants was evaluated using commercial DuoSet ELISA kit (R&D Systems) following the manufacturer’s instructions. A six-point standard curve using threefold serial dilutions and a high standard of 90,000 ng/mL was performed and run in replicate (coefficient of variation average 18 %). The accuracy of the methods was assessed by evaluating the agreement between the expected and measured values by Bland–Altman plot (all difference between repeated measures and expected values did not exceed 95 % confidence interval). Reliability of the test was estimated by Cronbach’s alpha coefficient (>0.99).

A four-parameter logistic (4-PL) curve-fit based on standard optic density values was used to calculate hyaluronan concentrations considering three decimals. The low molecular weight (15–40 kDa), medium molecular weight (75–350 kDa) and high molecular weight (>950 kDa) forms of Hyaluronan are all detected in this assay. These results were normalized for cell number and expressed as ng/10^6^ synoviocytes.

### Ethics committee approval

The study was approved by the Institutional Review Board and by Ethics Committee of Rizzoli Orthopedic Institute (ID no. 8342 of 2/04/2010). Written informed consent was signed by each subject.

### Statistical analysis

Data concerning the characterization of the different PRP preparations were analysed by Friedman’s test for multiple comparison of pared data and, when significant, followed by Bonferroni’s post hoc correction for multiple comparisons (value of *p* < 0.017 was considered significant after Bonferroni’s correction).

Results obtained by gene expression analysis and assessment of hyaluronic acid production were analysed by the general linear model (GLM).

Since data presented a skewed distribution, not fulfilling the hypothesis of normality, appropriate transformations were applied according to the following formula: *y* ^′^ = log _10_(*y* + 1). All the resulting data fulfilled the normality assumption as verified by the Kolmogorov–Smirnov test.

The GLM was used according to treatment condition (L-PRP, P-PRP, PPP), dose (5, 10, 20 %) and their combinations as fixed effects and the patient as a random effect. Partial Eta squared (η _p_^2^) was considered as evidence of the strength of the combination (effect size) between the fixed effects and gene expression levels of target molecules. The Sidak correction was applied for multiple comparisons. Value of *p* < 0.05 was considered significant.

Spearman’s correlation analysis was used to assess relationships between gene expression levels and platelet/leucocyte concentrations.

When GLM analysis was significant according to dose or treatment*dose association, the Kendall Tau correlation analysis was used to assess relationships between gene expression levels and doses of each preparation.

A value of *p* < 0.05 was considered significant.

Statistical analysis was carried out using SPSS v.19.0 (IBM Corp., Armonk, NY, USA) and GraphPad Prism for Windows (CA, USA).

## Results

### Characterization of the different PRP preparations

Platelet concentrations were significantly different among the three L-PRP, P-PRP and PPP preparations (Friedman’s Anova *p* < 0.001), and were also significantly different from one another (Bonferroni’s post hoc test, *p* < 0.017). White blood cells were present in L-PRP preparation, whereas they were almost undetectable in PPP and P-PRP (Table 2).

**Table 2:** Platelet and white blood cell concentrations in the different plasma preparations

	PPP	P-PRP	L-PRP
Platelet number/mm^3^	9,000 (6,500–11,500)	158,000 (83,000–343,000)	963,000 (793,000–995,000)
WBC/mm^3^	0	<200	5,400 (4,300–6,000)

Results are expressed as medians and (interquartile ranges) of seven donors

*PPP* platelet-poor plasma, *P-PRP* pure platelet-rich plasma, *L-PRP* leucocyte platelet-rich plasma, *WBC* white blood cells

In the different PRP and PPP preparations, excluding FGF-2 that showed similar releases among the preparations, IL-1β, HGF, PDGF AB/BB, TGF-β1, and VEGF concentrations were different (Friedman’s Anova *p* < 0.01, at least) (Table 3). In particular, IL-1β and VEGF concentrations were similar in PPP and P-PRP and both lower than those in the L-PRP preparation. HGF and TGF-β1 concentrations were lower in PPP than in P-PRP and L-PRP, but not significantly different between the two PRP preparations, whereas the PDGF AB/BB concentration progressively and significantly increased from PPP, to P-PRP, and then to L-PRP. All these factors showed increased or stable (PDGF AB/BB) concentrations after 7 days of incubation compared with those observed after 1 h of activation (not shown).

**Table 3:** Soluble factor concentrations in the different plasma preparations after 7 days of incubation

Soluble factors	PPP	P-PRP	L-PRP
IL-1β^b^	0.60 (0.02–3.06) *ns* versus *P*-*PRP* *p* < *0.017* versus *L*-*PRP*	0.45 (0.04–0.77) *p* < 0.017 versus *L*-*PRP*	60.7 (31.74–146.19)
FGF-2	4.53 (1.66–6.11) *ns* versus *P-PRP* and *L-PRP*	6.55 (0–13.77) *ns* versus *L*-*PRP*	13.22 (9.43–20.15)
HGF^c^	104.63 (41.88–135.78) *p* < *0.017* versus *P-PRP* and *L-PRP*	319.02 (150.83–366.23) *ns* versus *L*-*PRP*	392.26 (371.96–493.17)
PDGF AB/BB^b^	523.27 (239.19–1,210.48) *p* < *0.017* versus *P-PRP* and *L-PRP*	7,348.61 (1,008.00–10,113.68) *p* < *0.017* versus *L*-*PRP*	29,371.89 (17,555.47–81,690.26)
TGF-β1^c^	10,740.87 (2,408.46–11,896.05) *p* < *0.017* versus *P-PRP* and *L-PRP*	63,086.17 (15,632.91–640,603.53) *ns* versus *L*-*PRP*	103,553.39 (36,135.34–1,430,000.00)
VEGF^a^	3.73 (2.31–15.39) *ns* versus *P*-*PRP* *p* < *0.017* versus *L*-*PRP*	95.76 (67.76–161.95) *p* < *0.017* versus *L*-*PRP*	226.79 (181.49–771.13)

Concentrations (pg/ml) evaluated after 7 days of incubation are reported. Results are expressed as medians and (interquartile ranges) of seven donors

*PPP* platelet-poor plasma, *P-PRP* pure platelet-rich plasma, *L-PRP* leucocyte platelet-rich plasm, *ns* not significant *p* value

Comparisons among plasma preparations as determined by Friedman’s Anova test: ^a^ *p* < 0.01; ^b^ *p* < 0.005; ^c^ *p* < 0.002

### Gene expression analysis of factors involved in joint physiopathology

#### Pro-inflammatory factors, anti-inflammatory and/or anti-catabolic factors

**Fig. 1:**
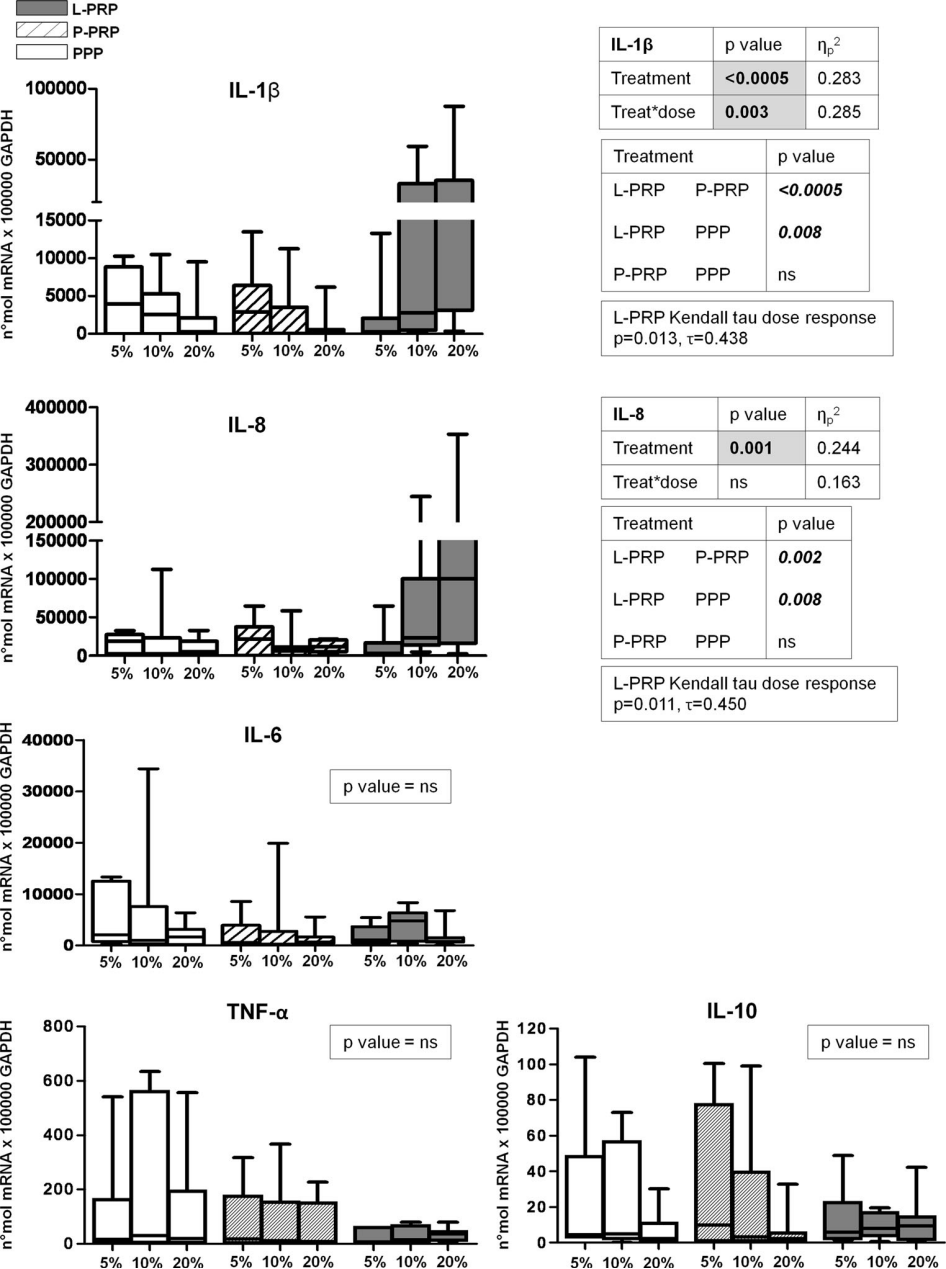
Gene expression analysis of interleukin (IL)-1beta, IL-8/CXCL8, IL-6, IL-10, tumour necrosis factor (TNF) alpha. Synovial fibroblasts were treated for 7 days with 5, 10, 20 % of L-PRP, P-PRP or PPP obtained from each subject (*n* = 7). Gene expression relative quantification was performed, and data are expressed as number of molecules *100,000 GAPDH. *Boxes* indicate the 25 and 75 % *percentiles, whiskers* indicate the minimum to maximum values, and *bars* indicate the median; *p* value significances are shown in tables beside each figure, as determined by General Linear Model statistical analysis and Kendall Tau correlation; *ns* not significant

Gene expression analysis indicated that IL-1β and IL-8/CXCL8 were significantly induced by L-PRP compared with both P-PRP and PPP (IL-1 β: *p* < 0.0005 L-PRP vs. P-PRP, *p* = 0.008 L-PRP vs. PPP; IL-8: *p* = 0.002 L-PRP vs. P-PRP, *p* = 0.008 L-PRP vs. PPP) (Fig. 1). Moreover, L-PRP also had a dose–response effect (Kendall Tau, IL-1β *p* = 0.013, IL-8 *p* = 0.011). Furthermore, if we consider the combined dose treatment effect on IL-1β gene expression, the dose–response curve showed a significantly different incremental trend induced by L-PRP from those of both P-PRP and PPP (*p* = 0.003) (Fig. 1). No significance between preparations was found on IL-6, IL-10 and TNF-α gene expression rate (Fig. 1), whereas IL-4 and IL-13 gene expression levels were not detectable in cultured synovial fibroblasts.

#### Growth factors, cartilage matrix-degrading enzymes and their inhibitors

**Fig. 2:**
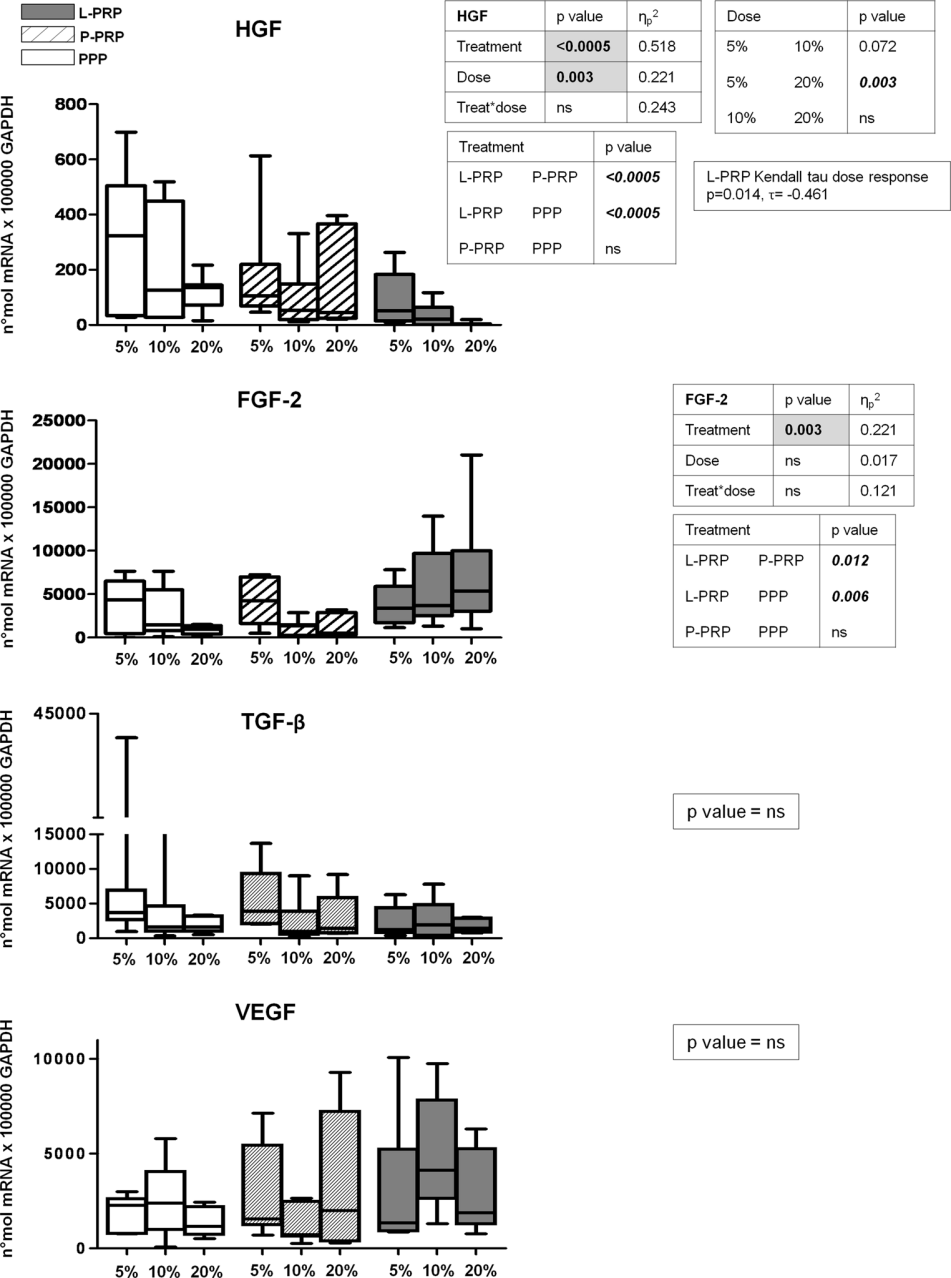
Gene expression analysis of hepatocyte growth factor (HGF), fibroblast growth factor (FGF)-2, transforming growth factor (TGF) beta, vascular endothelial growth factor (VEGF). Synovial fibroblasts were treated for 7 days with 5, 10, 20 % of L-PRP, P-PRP or PPP obtained from each subject (*n* = 7). Gene expression relative quantification was performed, and data are expressed as number of molecules *100,000 GAPDH. *Boxes* indicate the 25 and 75 % *percentiles*, *whiskers* indicate the minimum to maximum values, and *bars* indicate the median; *p* value significances are shown in tables beside each figure, as determined by General Linear Model statistical analysis and Kendall Tau correlation; *ns* not significant

Among GFs genes, VEGF and TGF-β were not differently modulated by the preparations (Fig. 2).

Interestingly, PRP preparations have an opposite effect on HGF and FGF-2 expression, due to their composition. Indeed, HGF expression is significantly down-modulated by L-PRP compared to both PRP and PPP (*p* < 0.0005 vs. P-PRP; *p* < 0.0005 vs. PPP). An inverse dose–response influence was shown for all preparations (5 vs. 20 % *p* = 0.003), which was particularly evident for L-PRP as confirmed by the Kendall Tau correlation (*p* = 0.014) (Fig. 2). Conversely, L-PRP significantly enhanced FGF-2 expression levels (*p* = 0.012 vs. P-PRP; *p* = 0.006 vs. PPP), with no dose contribution (Fig. 2). No differences were observed between P-PRP and PPP.

**Fig. 3:**
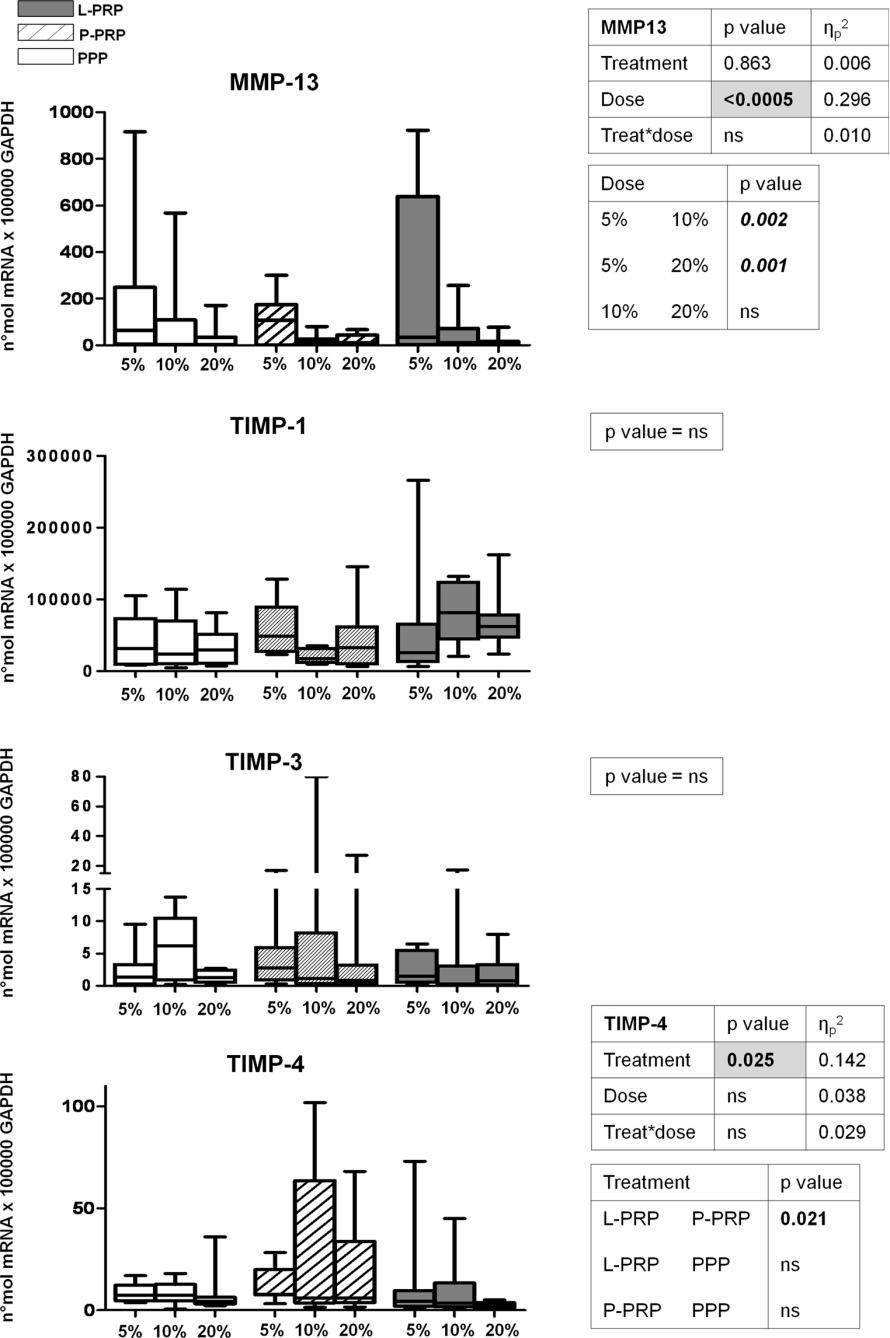
Gene expression analysis of cartilage matrix-degrading enzyme metalloproteinase-13 (MMP-13) and tissue inhibitor of metalloproteinase inhibitors (TIMP-1,-3,-4). Synovial fibroblasts were treated for 7 days with 5, 10, 20 % of L-PRP, P-PRP or PPP obtained from each subject (*n* = 7). Gene expression relative quantification was performed, and data are expressed as number of molecules *100,000 GAPDH. *Boxes* indicate the 25 and 75 % *percentiles*, *whiskers* indicate the minimum to maximum values, and *bars* indicate the median; *p* value significances are shown in tables beside each figure, as determined by General Linear Model statistical analysis; *ns* not significant

MMP-13 is one of the most important matrix-degrading enzymes and its expression appeared to be inversely related to concentration in all preparations, (5 vs. 10 % *p* = 0.002; 5 vs. 20 % *p* = 0.001) (Fig. 3). Concerning the tissue inhibitor of degrading matrix enzymes, TIMP-1, TIMP-3 and TIMP-4, only TIMP-4 was differently modulated by the PRP preparations, in particular L-PRP determined a statistically significant decrease in TIMP-4 expression compared to P-PRP (*p* = 0.021), with no dose or combined dose treatment effect (Fig. 3).

### HA production and HA synthases (HAS) gene expression

**Fig. 4:**
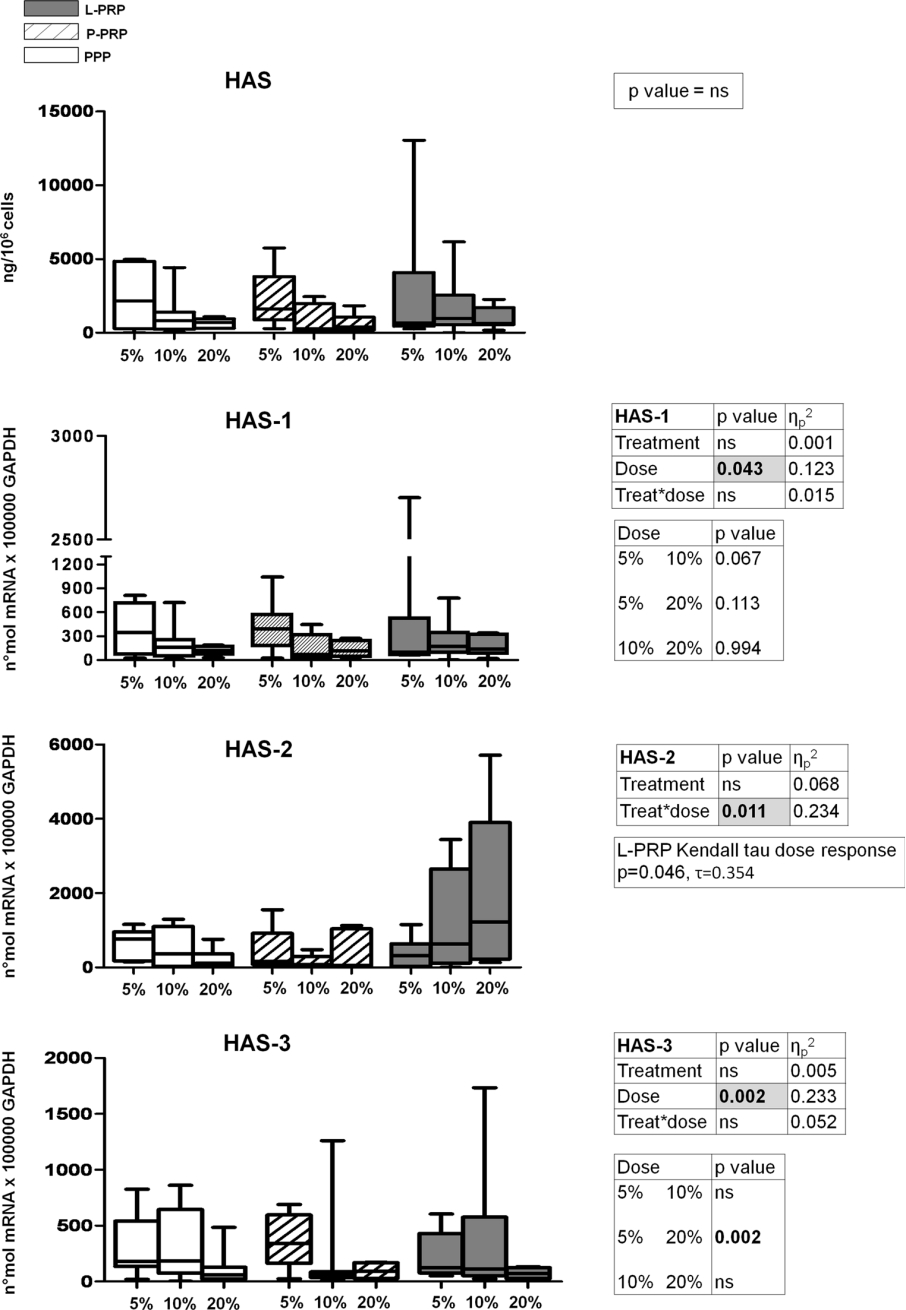
Hyaluronic acid modulation by PRP. Synovial fibroblasts were treated for 7 days with 5, 10, 20 % of L-PRP, P-PRP or PPP obtained from each subject (*n* = 7). Hyaluronic acid synthases 1-2-3 gene expression relative quantification was performed, and data are expressed as number of molecules *100,000 GAPDH. Hyaluronic acid protein production was normalized per number of cells. *Boxes* indicate the 25 and 75 % *percentiles*, *whiskers* indicate the minimum to maximum values, and *bars* indicate the median; *p* value significances are shown in tables beside each figure, as determined by General Linear Model statistical analysis and Kendall Tau correlation; *ns* not significant

No effect of the different preparations was observed on HA production or on gene expression of its synthases (Fig. 4). Despite this lack of a preparation-dependent effect, dose treatment trend of HAS-2 gene expression level stimulated by L-PRP was statistically different from that obtained by stimulating cells with P-PRP (*p* = 0.011) (Fig. 4); indeed, HAS-2 gene expression had an increasing course in the presence of L-PRP (significant correlation with doses was observed: Kendall Tau *p* = 0.014), whereas in the presence of P-PRP, it remained stable.

HAS-3 gene expression modulation seemed to have an inverse dose–response trend, not dependent on preparation type (5 vs. 20 % *p* = 0.0002) (Fig. 4).

### Correlations between gene expression level and white blood cells or platelet count

Both IL-1β and IL-8 expression levels were directly correlated with L-PRP white blood cell (WBC) count (IL-1 *p* = 0.0048, *r* = 0.59; IL-8/CXCL8 *p* = 0.0039, *r* = 0.60). HGF mRNA expression showed an inverse correlation with both WBC and platelet count (WBC *p* = 0.0293, *r* = −0.50; platelets *p* < 0.0001, *r* = −0.61). Interestingly, MMP-13 expression inversely correlated with L-PRP WBC content (*p* = 0.0331, *r* = −0.47). TIMP-4 inversely correlated with PRP platelet count (*p* = 0.0134, *r* = −0.31). HAS-2 and HAS-3 had, respectively, direct and inverse correlation trends with L-PRP WBC count (HAS-2 *p* = 0.0052, *r* = 0.59; HAS-3 *p* = 0.0327, *r* = −0.49) (not shown).

## Discussion

The main finding of the present study underlines that OA synovial fibroblasts appeared to be differentially modulated by L-PRP compared to P-PRP and PPP. Specifically, L-PRP was able to sustain a long-term up-regulation of IL-1β, IL-8/CXCL8 and FGF-2 gene expression levels compared to PRP and PPP. Conversely, a lower expression of TIMP-4 and HGF genes was found in the presence of L-PRP compared to either P-PRP or PPP.

Both IL-1β and IL-8/CXCL8 are well-recognized as pro-inflammatory agents, and their involvement in OA pathogenesis is extensively reported [for review see 7, 26, 28, 56]. The up-regulation of these genes induced by L-PRP might be ascribed to the most elevated levels reached by PDGF and TGF-β in L-PRP secretome, as previous studies reported that PDGF and TGF-β are able to synergistically potentiate IL-1β and IL-8/CXCL8 expression in OA synoviocytes [11, 12, 50]. Furthermore, since IL-1β is able to up-regulate both IL-8 and its own production, another possible explanation might be the presence of higher levels of IL-1β detected in L-PRP compared to those of P-PRP and PPP preparations, probably due to the WBC count. Indeed, IL-1β and IL-8 expression levels significantly correlate with WBC count and for both factors there is a dose–response effect.

Among the growth factors analysed in this study, FGF-β and HGF expressions were, respectively, up- and down-modulated by the L-PRP preparation, with a dose–response effect seen on HGF expression. Interestingly, FGF-2 and HGF seemed to exert opposite effects on OA cartilage: FGF-2 is considered to be a catabolic and anti-anabolic inducer in human cartilage [35, 59], whereas HGF has been shown to foster anti-inflammatory effects on human chondrocyte, by down-modulating Nuclear Factor kappa B [6], the main transcription factor regulating the inflammatory process. However, FGF-2 and HGF exert a wide spectrum of pleiotropic effects on OA cartilage and synovium, including pro-angiogenetic properties [36, 40]. The role of PRP in angiogenesis modulation is one of the main focuses of several studies. Angiogenesis may favour tissue repair, but it may also promote inflammation and the contribution of angiogenesis to joint modification has been extensively reported in OA [5, 38].

The present findings concerning HGF modulation in OA synoviocytes are in line with the results obtained by Anitua et al. [4], who described an inhibition of HGF production by fibroblasts exposed to a secretome from a high number of platelets. Conversely, since IL-1beta inhibited the OA synovial production of HGF [2], the lowest levels of expression reached by HFG in presence of L-PRP may also be due to the potential inhibitory effect of the IL-1beta present in the L-PRP preparation. Given the ability of WBC to produce IL-1 beta, this hypothesis is supported by evidence of the inverse correlation between HGF expression and WBC count.

Another key point of the present analysis is the effect of the different PRP preparations on specific enzymes that play a pivotal role in joint tissue remodelling: MMP-13, one of the most important matrix-degrading enzymes strongly involved in cartilage matrix breakdown and OA pathogenesis, and TIMP-1, TIMP-3, TIMP-4, tissue inhibitors of several matrix metalloproteinases able to counteract their degrading actions. Interestingly, MMP-13 was not differentially modulated by PRP preparations and PPP, in agreement with previously reported data concerning other MMPs, such as MMP-1 and MMP-3 [2].

Furthermore, a previous study [42] focused on tendon explant response treated with different PRP products, prepared according to an increasing concentration of leucocytes and different platelet/leucocyte ratios, the expression of MMP-13 was lower than that of the control group, in the presence of all PRP preparations although no differential expression of MMP-13 was found among the different preparations. The present results seem to be in line with these findings, since no differences were found between MMP-13 gene expression level between L-PRP and P-PRP stimulation. Unlike these authors, in the present study, no differences were found in MMP-13 expression between PPP and PRPs. This discrepancy might due to different reasons: first, the different cells tested in the present study (synovial tissue vs. tendon), given that tissue-specific response elicited by PRP has been highlighted in several studies [4, 41]; second, the different type of culture (isolated cells vs. explants) and third, the period of observation (7 days vs. 72 h).

These data together with the evidence that, in the present study, MMP-13 expression appeared to be inversely related to the increasing concentrations of the all different preparations (L-PRP, P-PRP, PPP) might support the hypothesis that MMP-13 gene regulation is mainly influenced by plasma proteome and/or by the ratio between platelet secretome and plasma proteins, as suggested by other authors [4], and not directly related to a single condition.

Concerning the TIMPs analysed, Anitua et al. [2] previously reported that platelet releasate appeared not to alter TIMP-1 production by OA synovial cells. Consistently with this finding, in the present study, TIMP-1 and TIMP-3 expression was not significantly modified by the different preparations, whereas a lower expression level of TIMP-4 was found in the presence of L-PRP compared with P-PRP.

Finally, because of the relevance of Hyaluronan in joint homoeostasis, as an important component of cartilage extracellular matrix and synovial fluid, another aim of the present study was to investigate the influence of PRP preparations on HA production by OA synoviocytes and on the expression of the different HAS isoforms. HA is synthesized at the plasma membrane by HAS, which are present as 3 transmembrane forms (HAS1-2-3) [30].

In the present study, no treatment regulation of HAS expression or HA production by the different PRP preparations or PPP was found, which is not in line with previously reported data [2]. This might be explained by the culture period. In fact these authors described a regulation of HA production after 72-h stimulation with PRP preparations, whereas the present authors maintained synoviocytes in culture for 7 days to reproduce the treatment schedule used in clinical practice, the effect of PRP on HA gene expression or production might no longer be visible after 7 days. Conversely, a different effect of dose treatment on HAS2 (L-PRP had an increased trend whereas P-PRP remained stable) and an inverse dose–response effect on HAS3 was noticed by the present authors (20 % dose reduced HAS3, not dependent on the type of PRP used). Although these enzymes catalyse the same reaction, they differ in the size of their products [30]. HAS-3 produces linear polymers of HA of smaller molecular sizes than those produced by HAS-1 and HAS-2. Furthermore, HAS-2 produces the largest molecules of the three isoforms. Therefore, L-PRP might play a role in reducing smaller molecular-sized polymers while enhancing larger molecular size hyaluronan. This effect might be beneficial because it is known that both the concentration and size of HA are reduced in OA synovial fluid [23] and that these small-sized HA molecules might have a proinflammatory effect in animal models [16].

Surprisingly, no differential effect was found on OA synoviocytes induced by P-PRP compared to PPP. These results might be ascribed to the lower concentrations of platelet secretome from P-PRP which might be insufficient to sustain a relevant modulation of gene expression up to 7 days.

Taking into account the pattern of molecules modulated by L-PRP and their role in joint homoeostasis, the overall results that emerge from this study highlight that the net effect of L-PRP might prompt an inflammatory activation of OA synoviocytes, given the ability of this preparation to induce, for at least 7 days, an enhancement of proinflammatory and procatabolic factors such as IL-1beta, IL-8, and FGF-2 together with a lowering of TIMP-4 expression.

These results added to the evidence of a significant correlation between leucocyte number and both IL-1 expression and IL-8 expression, together with the finding of a significantly different dose–response trend observed for IL-1 expression in the presence of L-PRP might support the hotly debated hypothesis that leucocytes in PRP might foster unwanted effects.

The potentiality of L-PRP preparation to induce pro-inflammatory events has been reported by other authors, both in human and animal model “in vitro” studies [10, 42].

Interestingly, a clinical study, recently published [21], has underlined that the presence of leucocytes in the “double-spinning” preparation does not seem to influence the therapeutic efficacy of PRP in the treatment of cartilage degeneration and OA, even if the occurrence of minor adverse events (swelling and pain) were more frequently reported in this group of patients.

The results obtained in the aforementioned clinical study might be partially related to the findings of the present study, but this assertion remains a mere speculation, given the limitations of “in vitro” tissue-specific studies that cannot mirror the complexity of joint environment.

Another potential limitation of this study arise by the consideration that, even if the majority of research studies address the pathophysiology of synovial tissue focusing on fibroblast-like synoviocytes, additional relevant cell types, including monocytes, macrophages, T and B cells, are present in synovium and actively and collectively operate modulating the joint response [8, 53].

Further researches are needed to clarify the influence of the different PRP components (platelets and leucocytes) and their concentration on the bioactivity of PRP. Since leucocyte–platelet interaction may promote the biosynthesis of other factors that facilitate the resolution of inflammation, such as lipoxins [31], and given their involvement in immune-response [45], the optimization of their concentrations in PRP products might lead to minimizing or avoiding the detrimental effects ascribed to leucocytes and exploiting their beneficial properties.

As for clinical relevance, the evaluation of the optimal cell concentrations and their ratio (relative proportions) in PRP will be important issues for future directions.

Further studies need to focus on the contribution of PRP components to the overall observed clinical outcome, helping to develop concentrates with specific and desired effects.

## Conclusion

The present data on OA synoviocytes indicate, on one hand, that, at 7 days, P-PRP was not able to exert a differential pattern of biological effect compared to PPP, and, on the other hand, that L-PRP is able to sustain long-term up-regulation of proinflammatory factors, such as IL-1beta, IL-8 and FGF-2, together with a down-modulation of HGF and TIMP-4 expression, two factors that have been recognized as anti-catabolic mediators in cartilage, thus supporting the need to further optimize PRP preparations to be applied in the clinical practice.

## References

[1] Ahmad Z, Howard D, Brooks RA, Wardale J, Henson FM, Getgood A, Rushton N (2012). The role of platelet rich plasma in musculoskeletal science. JRSM Short Rep.

[2] Anitua E, Sanchez M, Nurden AT, Zalduendo MM, de la Fuente M, Azofra J, Andia I (2007). Platelet-released growth factors enhance the secretion of hyaluronic acid and induce hepatocyte growth factor production by synovial fibroblasts from arthritic patients. Rheumatology (Oxford).

[3] Anitua E, Orive G, Aguirre JJ, Andia I (2008). Clinical outcome of immediately loaded dental implants bioactivated with plasma rich in growth factors: a 5-year retrospective study. J Periodontol.

[4] Anitua E, Sanchez M, Zalduendo MM, de la Fuente M, Prado R, Orive G, Andia I (2009). Fibroblastic response to treatment with different preparations rich in growth factors. Cell Prolif.

[5] Ashraf S, Walsh DA (2008). Angiogenesis in osteoarthritis. Curr Opin Rheumatol.

[6] Bendinelli P, Matteucci E, Dogliotti G, Corsi MM, Banfi G, Maroni P, Desiderio MA (2010). Molecular basis of anti-inflammatory action of platelet-rich plasma on human chondrocytes: mechanisms of NF-kappa B inhibition via HGF. J Cell Physiol.

[7] Berenbaum F (2013). Osteoarthritis as an inflammatory disease (osteoarthritis is not osteoarthrosis!). Osteoarthr Cartil.

[8] Bondeson J, Wainwright SD, Lauder S, Amos N, Hughes CE (2006). The role of synovial macrophages and macrophage-produced cytokines in driving aggrecan ases, matrix metalloproteinases, and other destructive and inflammatory responses in osteoarthritis. Arthritis Res Ther.

[9] Braun HJ, Kim HJ, Chu CR, Dragoo JL (2014). The effect of platelet-rich plasma formulations and blood products on human synoviocytes: implications for intra-articular injury and therapy. Am J Sports Med.

[10] Browning SR, Weiser AM, Woolf N, Golish SR, SanGiovanni TP, Scuderi GJ, Carballo C, Hanna LS (2012). Platelet-rich plasma increases matrix metalloproteinases in cultures of human synovial fibroblasts. J Bone Joint Surg Am.

[11] Cheon H, Yu SJ, Yoo DH, Chae IJ, Song GG, Sohn J (2002). Increased expression of pro-inflammatory cytokines and metalloproteinase-1 by TGF-beta1 in synovial fibroblasts from rheumatoid arthritis and normal individuals. Clin Exp Immunol.

[12] Cheon H, Sun YK, Yu SJ, Lee YH, Ji JD, Song GG, Lee JH, Kim MK, Sohn J (2004). Platelet-derived growth factor-AA increases IL-1beta and IL-8 expression and activates NF-kappaB in rheumatoid fibroblast-like synoviocytes. Scand J Immunol.

[13] Cole BJ, Seroyer ST, Filardo G, Bajaj S, Fortier LA (2010). Platelet-rich plasma: where are we now and where are we going?. Sports Health.

[14] David-Raoudi M, Deschrevel B, Leclercq S, Galera P, Boumediene K, Pujol JP (2009). Chondroitin sulfate increases hyaluronan production by human synoviocytes through differential regulation of hyaluronan synthases: role of p38 and Akt. Arthritis Rheum.

[15] Dohan Ehrenfest DM, Rasmusson L, Albrektsson T (2009). Classification of platelet concentrates: from pure platelet-rich plasma (P-PRP) to leucocyte- and platelet-rich fibrin (L-PRF). Trends Biotechnol.

[16] Campo GM, Avenoso A, D’Ascola A (2012). Hyaluronan differently modulates TLR-and the inflammatory response in mouse chondrocytes. Biofactors.

[17] Engebretsen L, Steffen K, Alsousou J, Anitua E, Bachl N, Devilee R, Everts P, Hamilton B, Huard J, Jenoure P, Kelberine F, Kon E, Maffulli N, Matheson G, Mei-Dan O, Menetrey J, Philippon M, Randelli P, Schamasch P, Schwellnus M, Vernec A, Verrall G (2010). IOC consensus paper on the use of platelet-rich plasma in sports medicine. Br J Sports Med.

[18] Filardo G, Kon E, Buda R, Timoncini A, Di Martino A, Cenacchi A, Fornasari PM, Giannini S, Marcacci M (2011). Platelet-rich plasma intra-articular knee injections for the treatment of degenerative cartilage lesions and osteoarthritis. Knee Surg Sports Traumatol Arthrosc.

[19] Filardo G, Kon E, Di Martino A, Di Matteo B, Merli ML, Cenacchi A, Fornasari PM, Marcacci M (2012). Platelet-rich plasma vs hyaluronic acid to treat knee degenerative pathology: study design and preliminary results of a randomized controlled trial. BMC Musculoskelet Disord.

[20] Filardo G, Kon E (2012). PRP: more words than facts. Knee Surg Sports Traumatol Arthrosc.

[21] Filardo G, Kon E, Pereira Ruiz MT, Vaccaro F, Guitaldi R, Di MA, Cenacchi A, Fornasari PM, Marcacci M (2012). Platelet-rich plasma intra-articular injections for cartilage degeneration and osteoarthritis: single- versus double-spinning approach. Knee Surg Sports Traumatol Arthrosc.

[22] Filardo G, Kon E, Roffi A, Di Matteo B, Merli ML, Marcacci M (2013) Platelet-rich plasma: why intra-articular? A systematic review of preclinical studies and clinical evidence on PRP for joint degeneration. Knee Surg Sports Traumatol Arthrosc. doi:10.1007/s00167-013-2743-1 10.1007/s00167-013-2743-1 PMC4541701 24275957

[23] Flugge LA, Miller-Deist LA, Petillo PA (1999). Towards a molecular understanding of arthritis. Chem Biol.

[24] Foster TE, Puskas BL, Mandelbaum BR, Gerhardt MB, Rodeo SA (2009). Platelet-rich plasma: from basic science to clinical applications. Am J Sports Med.

[25] Gobbi A, Karnatzikos G, Mahajan V, Malchira S (2012). Platelet-rich plasma treatment in symptomatic patients with knee osteoarthritis: preliminary results in a group of active patients. Sports Health.

[26] Goldring MB, Otero M (2011). Inflammation in osteoarthritis. Curr Opin Rheumatol.

[27] Hall MP, Band PA, Meislin RJ, Jazrawi LM, Cardone DA (2009). Platelet-rich plasma: current concepts and application in sports medicine. J Am Acad Orthop Surg.

[28] Haseeb A, Haqqi TM (2013). Immunopathogenesis of osteoarthritis. Clin Immunol.

[29] Hochberg MC, Altman RD, Brandt KD, Clark BM, Dieppe PA, Griffin MR, Moskowitz RW, Schnitzer TJ (1995). Guidelines for the medical management of osteoarthritis: part II. Osteoarthritis of the knee. American college of rheumatology. Arthritis Rheum.

[30] Itano N, Sawai T, Yoshida M, Lenas P, Yamada Y, Imagawa M, Shinomura T, Hamaguchi M, Yoshida Y, Ohnuki Y, Miyauchi S, Spicer AP, McDonald JA, Kimata K (1999). Three isoforms of mammalian hyaluronan synthases have distinct enzymatic properties. J Biol Chem.

[31] Kantarci A, Van Dyke TE (2003). Lipoxins in chronic inflammation. Crit Rev Oral Biol Med.

[32] Kellgren JH, Lawrence JS (1957). Radiological assessment of osteo-arthrosis. Ann Rheum Dis.

[33] Kon E, Filardo G, Drobnic M, Madry H, Jelic M, van Dijk N, Della Villa S (2012). Non-surgical management of early knee osteoarthritis. Knee Surg Sports Traumatol Arthrosc.

[34] Kon E, Filardo G, Matteo BD, Marcacci M (2013). PRP for the treatment of cartilage pathology. Open Orthop J.

[35] Li X, Ellman MB, Kroin JS, Chen D, Yan D, Mikecz K, Ranjan KC, Xiao G, Stein GS, Kim SG, Cole B, van Wijnen AJ, Im HJ (2012). Species-specific biological effects of FGF-2 in articular cartilage: implication for distinct roles within the FGF receptor family. J Cell Biochem.

[36] Lin YM, Huang YL, Fong YC, Tsai CH, Chou MC, Tang CH (2012). Hepatocyte growth factor increases vascular endothelial growth factor-A production in human synovial fibroblasts through c-Met receptor pathway. PLoS One.

[37] Liu-Bryan R (2013). Synovium and the innate inflammatory network in osteoarthritis progression. Curr Rheumatol Rep.

[38] Mapp PI, Walsh DA (2012). Mechanisms and targets of angiogenesis and nerve growth in osteoarthritis. Nat Rev Rheumatol.

[39] Mariani E, Cattini L, Neri S, Malavolta M, Mocchegiani E, Ravaglia G, Facchini A (2006). Simultaneous evaluation of circulating chemokine and cytokine profiles in elderly subjects by multiplex technology: relationship with zinc status. Biogerontology.

[40] Maruotti N, Cantatore FP, Crivellato E, Vacca A, Ribatti D (2006). Angiogenesis in rheumatoid arthritis. Histol Histopathol.

[41] Mazzocca AD, McCarthy MB, Chowaniec DM, Dugdale EM, Hansen D, Cote MP, Bradley JP, Romeo AA, Arciero RA, Beitzel K (2012). The positive effects of different platelet-rich plasma methods on human muscle, bone, and tendon cells. Am J Sports Med.

[42] McCarrel TM, Minas T, Fortier LA (2012). Optimization of leukocyte concentration in platelet-rich plasma for the treatment of tendinopathy. J Bone Joint Surg Am.

[43] Moojen DJ, Everts PA, Schure RM (2008). Antimicrobial activity of platelet-leukocyte gel against staphylococcus aureus. J Orthop Res.

[44] Murphy L, Schwartz TA, Helmick CG, Renner JB, Tudor G, Koch G, Dragomir A, Kalsbeek WD, Luta G, Jordan JM (2008). Lifetime risk of symptomatic knee osteoarthritis. Arthritis Rheum.

[45] Nurden AT (2011). Platelets, inflammation and tissue regeneration. Thromb Haemost.

[46] Park SI, Lee HR, Kim S, Ahn MW, Do SH (2012). Time-sequential modulation in expression of growth factors from platelet-rich plasma (PRP) on the chondrocyte cultures. Mol Cell Biochem.

[47] Patel S, Dhillon MS, Aggarwal S, Marwaha N, Jain A (2013). Treatment with platelet-rich plasma is more effective than placebo for knee osteoarthritis: a prospective, double-blind, randomized trial. Am J Sports Med.

[48] Perut F, Filardo G, Mariani E, Cenacchi A, Pratelli L, Devescovi V, Kon E, Marcacci M, Facchini A, Baldini N, Granchi D (2013). Preparation method and growth factor content of platelet concentrate influence the osteogenic differentiation of bone marrow stromal cells. Cytotherapy.

[49] Poole AR (2012). Osteoarthritis as a whole joint disease. HSS J.

[50] Rosengren S, Corr M, Boyle DL (2010). Platelet-derived growth factor and transforming growth factor beta synergistically potentiate inflammatory mediator synthesis by fibroblast-like synoviocytes. Arthritis Res Ther.

[51] Sanchez M, Fiz N, Azofra J, Usabiaga J, Aduriz RE, Garcia GA, Albillos J, Garate R, Aguirre JJ, Padilla S, Orive G, Anitua E (2012). A randomized clinical trial evaluating plasma rich in growth factors (PRGF-Endoret) versus hyaluronic acid in the short-term treatment of symptomatic knee osteoarthritis. Arthroscopy.

[52] Scanzello CR, Goldring SR (2012). The role of synovitis in osteoarthritis pathogenesis. Bone.

[53] Sellam J, Berenbaum F (2010). The role of synovitis in pathophysiology and clinical symptoms of osteoarthritis. Nat Rev Rheumatol.

[54] Smyth NA, Murawski CD, Fortier LA, Cole BJ, Kennedy JG (2013). Platelet-rich plasma in the pathologic processes of cartilage: review of basic science evidence. Arthroscopy.

[55] Spakova T, Rosocha J, Lacko M, Harvanova D, Gharaibeh A (2012). Treatment of knee joint osteoarthritis with autologous platelet-rich plasma in comparison with hyaluronic acid. Am J Phys Med Rehabil.

[56] Sun HB (2010). Mechanical loading, cartilage degradation, and arthritis. Ann NY Acad Sci.

[57] Tsay RC, Vo J, Burke A, Eisig SB, Lu HH, Landesberg R (2005). Differential growth factor retention by platelet-rich plasma composites. J Oral Maxillofac Surg.

[58] Tschon M, Fini M, Giardino R, Filardo G, Dallari D, Torricelli P, Martini L, Giavaresi G, Kon E, Maltarello MC, Nicolini A, Carpi A (2011). Lights and shadows concerning platelet products for musculoskeletal regeneration. Front Biosci (Elite Ed).

[59] Vincent TL (2011). Fibroblast growth factor 2: good or bad guy in the joint?. Arthritis Res Ther.

[60] Wang-Saegusa A, Cugat R, Ares O, Seijas R, Cusco X, Garcia-Balletbo M (2011). Infiltration of plasma rich in growth factors for osteoarthritis of the knee short-term effects on function and quality of life. Arch Orthop Trauma Surg.

